# Effect of Vegetable Oil on the Properties of Asphalt Binder Modified with High Density Polyethylene

**DOI:** 10.3390/polym15030749

**Published:** 2023-02-01

**Authors:** Elizabeth Langa, Giovanna Buonocore, Antonino Squillace, Herminio Muiambo

**Affiliations:** 1 Department of Chemistry, Faculty of Sciences, Eduardo Mondlane University, Maputo P.O. Box 257, Mozambique; 2 Institute of Polymers, Composites and Biomaterials, National Research Council, P.le E. Fermi, 1, 80055 Naples, Italy; 3 Materials and Industrial Production, Department of Chemical Engineering, University of Naples Federico II, P.le Tecchio, 80125 Naples, Italy

**Keywords:** asphalt, vegetable oil, soybean, HDPE, rheology

## Abstract

Economic development results in increased traffic and higher traffic loads that often cause serious asphalt pavement problems, such as permanent deformation, fatigue cracking, and reduced lifetime. Polymers are seen as viable asphalt additives to minimize these problems. However, their incorporation reduces the workability of the material due to the increase in the viscosity of the blend. This study evaluates the effect of the addition of soybean oil on the physical, rheological, and thermal properties of high-density polyethylene (HDPE)-modified asphalt binder. The HDPE was kept at 5 wt.% and the soybean oil the asphalt was varied from 1 to 7 wt.%. A series of tests was conducted to evaluate the binders, comprising conventional tests (penetration, softening point, and ductility) rheological performance tests (dynamic viscosity and short-term aging (RTFO), and thermogravimetric analysis (TGA). The addition of HDPE reduced the penetration and increased the softening point and viscosity. The oil reduced steadily the viscosity, improved the workability and the thermal susceptibility of the modified asphalt up to 3 wt.% of oil, and reduced about 92% mass gain after aging. Hence, the oil is considered a good modifier agent for the improvement of polymer-modified asphalt’s workability under service conditions.

## 1. Introduction

Asphalt is a viscoelastic material produced as a by-product during the thermal decomposition of organic substances. Its most sought-after property in road construction is the ability to adhere very well to an aggregate surface and maintain the integrity of the road surface during its useful life [1]. When exposed to air and water, the asphalt undergoes chemical transformations that lead to an aging process by oxidation. This is a natural phenomenon that starts in the phase of construction of the hot-executed asphalt layers and develops over the lifetime of the asphalt pavement [2]. The main consequence of this aging phenomenon is the hardening that is expressed by the increase in the complex module, which decreases asphalt permanent deformation resistance in the first months after construction [3,4]. The search for high-quality asphalt and the limitations of the refining process are driving research into asphalt binders reinforced with polymers. As a way of guaranteeing the performance of asphalt in different environments and improving or promoting its properties, virgin and recycled materials have been used, which include styrene-butadiene-styrene (SBS), styrene-butadiene rubber (SBR), crumb rubber (CR), polyphosphoric acid (PPA), recycled polyethylene (PE), residual wood derived from bio-oil, plastic waste, residual cooking oil, and residual motor oil [5,6,7,8,9]. Some attempts have been looking at increasing the lifetime of paved roads by applying polymeric waste [10,11] and vegetable oils [12,13,14] as asphalt additives.

Several authors have demonstrated that the viscoelasticity and thermal susceptibility of asphalt can be improved by the use of additives, especially polymers [15,16,17]. According to Becker & Méndez [18], the appropriate addition of polymers can improve the quality of asphalt. Polyethylene-based modification for asphalt is increasingly used in paving engineering as a response to the growing demand for better pavement performance. The formation of cracks in asphalt pavements can reduce the useful lifetime and jeopardize the safety of road users. In this sense, high-density polyethylene (HDPE) is one of the thermoplastics that can be used to modify the agglutinating asphalt and reduce permanent deformation as well as to improve asphalt and asphalt mixing properties [19,20]. The internal structure of polyethylene and the resulting asphalt are of great interest due to their major influence on the performance of the pavement. They show improved rutting resistance of asphalt concrete, improved mechanical properties of the binders at high temperatures, and better resistance against permanent deformation [21]. On the other hand, the addition of polymers to asphalt leads to an increase in viscosity, which leads to operational complications that are primarily related to mixing and storage [22,23]. The low compatibility between asphalt and polymer can lead to phase separation when the material is stored at high temperatures (160–200 °C) in the absence of agitation [24]. Appiah et al. [10] modified the asphalt with HDPE and polypropylene (PP) and observed that these polymers increase the asphalt viscosity and consequently increase the processing temperature of the modified asphalt. In order to reduce the viscosity of the asphalt and improve its workability, various additives such as recycled vegetable oils and engine oil have been suggested [9,12,20,21]. A study carried out by Pereira et al. [25] revealed that in recent years, there has been also greater interest in the application of vegetable oils as modifiers for asphalt binders. Haghshenas et al. [8] evaluated the effects of aromatic extracts, paraffinic oil, naphthenic oil, triglycerides/fatty acids, and tall oil on the resistance to cracking associated with aging and the resistance to moisture damage of modified asphalt binders. Their results showed that, unlike aromatic extracts, the triglyceride/fatty acid and tall oil recycling agents that contain oxygen and carbonyl and hydroxyl functional groups could not maintain long-term low-temperature cracking resistance after aging, similar to the results reported by Zaumanis et al. [26]. According to Zaumanis et al. [26], the use of waste vegetable oil, waste vegetable grease, organic oil, waste engine oil, and aromatic extracts as recycling agents increases the cracking susceptibility of reclaimed asphalt pavement mixtures and decreases the low-temperature cracking resistance.

Results obtained by Dong et al. [27] demonstrated that waste engine oil and waste cooking oil could soften and recover the workability of aged asphalt effectively. Meanwhile, Portugal [28] studied the effect of the incorporation of new and residual soybean and corn oil into the asphalt and verified that the use of soybean oil and corn oil (new and residual) decreases the viscosity of the mixtures with a consequent decrease in the machining temperature and compaction of the mixtures. He also observed that there are no significant differences in the use of new or residual oil. In addition, Costa et al. [29] evaluated the mechanical properties of modified asphalt mixtures prepared with recycled soybean oil and corn oil and observed reduction of the asphalt mixtures maximum strength, stiffness, and resistance to permanent deformation regardless of the use of new or residual oil (advantageous from the environmental impact point of view). Currently, many researchers, while trying to improve the quality of asphalt, have also aimed to reduce the level of pollution caused by the inappropriate disposal of polymeric waste and residual vegetable oil.

Many researchers have carried out various laboratory experiments related to the effect of HDPE and vegetable oils on the characteristics of the modified binders; however, few experimental studies have been carried out to evaluate the effect of simultaneous modification of asphalt using HDPE and vegetable oil. In this research, the effect of HDPE and new soybean vegetable oil on the asphalt binders physical, rheological, and thermal properties was investigated through the following tests: penetration, ring and ball, viscosity, ductility, RTFO (rolling thin-film oven) aging, and thermogravimetric analysis (TGA). These tests provide valuable insights regarding the modified asphalt binder’s performance during the compaction and application stages.

## 2. Materials and Methods

### 2.1. Materials

The asphalt used in this study is classified as 50/70 penetration grade, supplied by Puma Energy Mozambique. Its properties are shown in Table 1. The HDPE powder was supplied by Sasol SA, has a density of 0.95 g/cm^3^, a melt flow index (MFI) of 1.70 g/10 min at 190 °C, a melting temperature between 130–135 °C, and an average particle diameter of 0.80 mm. The soybean vegetable oil “Fló” was supplied by the local company Maeva Matola, Mozambique.

### 2.2. Preparation of Modified Asphalt by Wet Modification Method

Initially, in a stainless steel vessel, neat asphalt was heated and stirred until melting and kept at a temperature range of 130–135 °C, at 5000 rpm in a high shear mixer, IKA^®^ EUROSTAR 20 for 20 min [10]. Then, 5 wt.% HDPE polymer powder was gradually added and heated to 170 °C at 5000 rpm for 20 more min. The HDPE concentration was kept constant at 5 wt.% and the soybean vegetable oil content was varied. It was gradually added in the proportions indicated in Table 2. The mixture was kept at 160 °C–170 °C at a constant stirring speed (5000 rpm) for another 15 min, and a homogenous mixture was obtained. The control sample (neat asphalt) was also submitted to the same protocol. Then, the blends were placed in small containers for further analysis.

**Figure 1:**
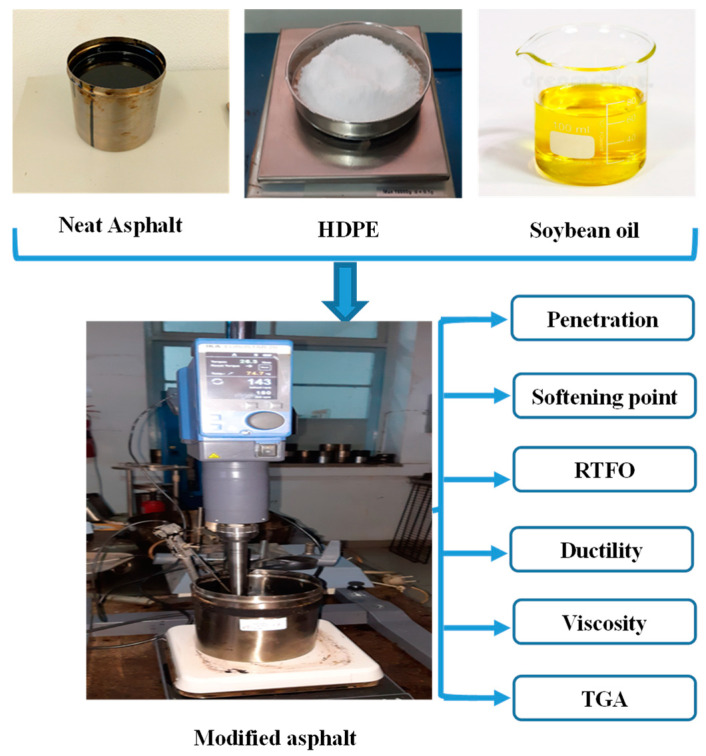
Experimental flowchart followed for preparation and characterization of asphalt samples.

The diagram below illustrates the experimental part (Figure 1).

A total of 6 samples, with different compositions, were obtained from the mixture of asphalt, polymer, and soybean vegetable oil. For all tests, triplicates of each sample were analyzed and the mean and standard deviation were determined, except for thermogravimetric analysis (TGA), where single runs were considered.

### 2.3. Test Methods

#### 2.3.1. Conventional Physical Tests

The penetration test was performed at 25 °C, with a load weight of 100 g and a needle penetration time of 5 s, according to the ASTM D5 [30]. The ring and ball softening point of asphalt material was carried out according to the ASTM D36 and used to estimate the thermal susceptibility of the asphalt [31]. The viscosity test at 165 °C was conducted following the procedures set out in the ASTM D4402 [32] to determine asphalt flow at high temperatures as well as the measurement of asphalt workability. The ductility at 25 °C was performed following the ASTM D113 standard [33].

The thermal susceptibility index, also called the penetration index (PI), was used to study the sensitivity of asphalt at different temperatures. The PI was evaluated by the procedure proposed (in 1936) by Pfeiffer and Van Doormaal, from the softening point (SP) of the petroleum asphalt and its penetration (Pen) at 25 °C, as shown in Equation (1) [34].

(1) $$\mathrm{PI}=\frac{1952-500\mathrm{logPen}-20\mathrm{SP}}{50\mathrm{logPen}-\mathrm{SP}-120}$$

#### 2.3.2. Short-Term Aging Test

The rotating thin-film oven (RTFO) test was carried out at 163 °C and the weight variation was determined. The test was performed according to the procedures described in the ASTM D2872 [35]. The samples were aged in a RTFO, and after the short-term aging test, the samples were again subjected to viscosity and ductility tests. The mass variation of the samples was determined using Equation (2):

(2) $$M=\frac{M_{\mathrm{initial}}-M_{\mathrm{final}}}{M_{\mathrm{initial}}}\;\times \;100\;$$

where M implies change in mass (%); *M*_initial_ is mass of asphalt before RTFO (g); and *M*_final_ represents the mass of asphalt after RTFO (g);

The antiaging property of asphalt was evaluated by the viscosity index (VAI) and ductility aging ratio (DAR), according to Equations (3) and (4). The greater the VAI and DAR, the more serious the aging of asphalt [36].

(3) $$\mathrm{DAR}=\frac{D_{\mathrm{after}}}{D_{\mathrm{before}}}\times 100\;$$

(4) $$\mathrm{VAI}=\frac{V_{\mathrm{after}}-V_{\mathrm{before}}}{V_{\mathrm{before}}}\times 100\;$$

where *V*_before_ and *D*_before_ are viscosity and ductility before RTFO, and *V*_after_ and *D*_after_ are their respective viscosity and ductility after RTFO.

#### 2.3.3. Thermogravimetric Analysis (TGA)

In this study, the thermal behavior of the asphalt and its composites was studied with the Q600 Shimadzu TGA instrument. Tests were performed in a nitrogen (N_2_) atmosphere (to rule out oxidation by atmospheric oxygen). About 15 ± 0.1 mg of sample was analyzed at a heating rate of 10 °C/min, from 25 to 600 °C.

## 3. Results and Discussion

### 3.1. Penetration

**Figure 2:**
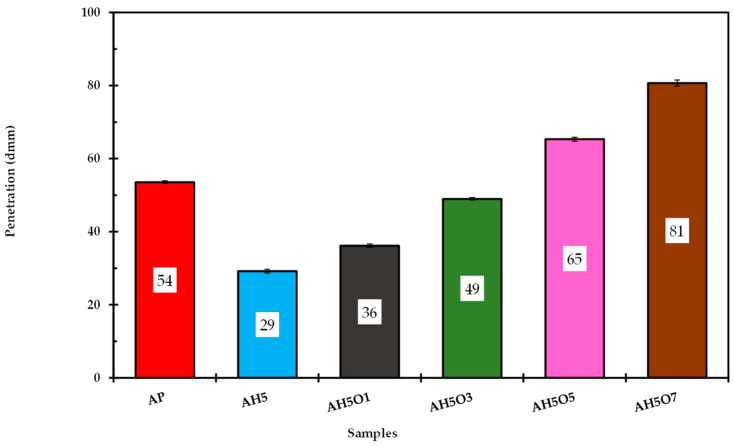
Penetration test results determined by the conventional method.

The asphalt binder penetration value represents consistency, reflecting the rheological properties of asphalt and describing the flow and deformation properties of binders. The effect of soybean vegetable oil on the HDPE-modified asphalt penetration value is shown in Figure 2. These results illustrate that HDPE decreases penetration of the neat asphalt binders. At 5 wt.% HDPE content, it was observed that the penetration decreases by almost 45% compared with unmodified binder. This result indicates greater rigidity of the mixture obtained and consequently a more resistant pavement to traffic loads. A similar result was also reported by Appiah [10]. The penetration value is inversely proportional to the hardness of the asphalt. The addition of polymer leads to an increase in the hardness of the asphalt, which can lead to an improvement in the permanent deformation of the asphalt [37,38]. The use of HDPE increases the stiffness of the asphalt mix which reveals greater resistance to cracking, especially in hot climate areas [20].

It was also noticeable that HDPE has a strong effect on neat binder as indicated through reduction of the penetration values and consequent increase of the stiffness, similar to the observations of Bala et al. [39]. The intense reduction of the penetration was due to diffusion of the oil fraction within the maltenes in the polymeric phase, which causes higher interactions and swelling between the HDPE polymer modifier and asphaltenes (polar molecules of the binder).

By contrast, with the incorporation of soybean oil, the penetration of HDPE-modified asphalt increased sharply with the increase in the soybean oil content from 1 to 7 wt.%. The addition of 1, 3, 5, and 7 wt.% of oil increased the HDPE-modified asphalt penetration by 24, 69, 124, and 179%, respectively. According to Rasman et al. [40], the high penetration values lead to decreasing hardness, thus producing binders with superior improvement for cracking resistance performance at low temperatures. These improvements can be explained by the natural fluidity characteristic of cooking oil. However, at higher temperatures, the binders with higher cooking oil content are expected to have poor performance in permanent deformation resistance since higher penetration binders are soft and unable to withstand high temperature exposure [41]. Thus, choosing an appropriate content of cooking oil is key to optimizing the high-temperature performance of the composite modified asphalt.

### 3.2. Softening Point

**Figure 3:**
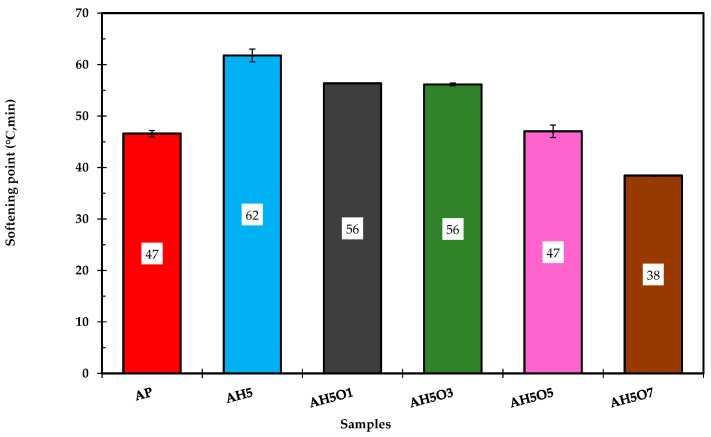
Softening point test results of the asphalt binders.

The softening point defines the plastic flow of asphalt and reflects the high temperature stability of the asphalt. Generally, the higher the softening point, the more stable the asphalt is at high temperatures [39,42]. From Figure 3, compared with unmodified asphalt binder, the softening point of asphalt/HDPE composite is increased by 15 °C, which indicates improvements in creep resistance [17,37,43]. The high strength of the polymers at elevated temperatures, compared to neat asphalt, is the reason behind the increase in the softening point of the resulting mixture of asphalt/HDPE composite. However, the addition of soybean oil decreased the softening point of the asphalt/HDPE/oil continually. As stated by Xu et al. [40], a higher value of asphalt softening point describes the higher stability of the binder under high service temperatures for paving applications. On the other hand, ternary composites with higher soybean oil content were more susceptible to temperature variation and less resistant to permanent deformation.

The oils soften the asphalt by reducing the content of high-molecular-mass asphaltenes and increasing the content of low-molecular-mass maltenes present in the asphalt [17,44,45,46]. From the penetration and softening point tests results, it was noticeable that compositions with more than 3 wt.% of soybean oil lead to poor performance at high temperatures.

The sample AH5O7, prepared with 7 wt.% soybean oil, showed a relatively low softening point and high penetration value. This sample softens at 38 °C, temperatures normally recorded in tropical climate countries and well below the peak temperatures reached in summer (45 °C).

### 3.3. Thermal Susceptibility Index

**Figure 4:**
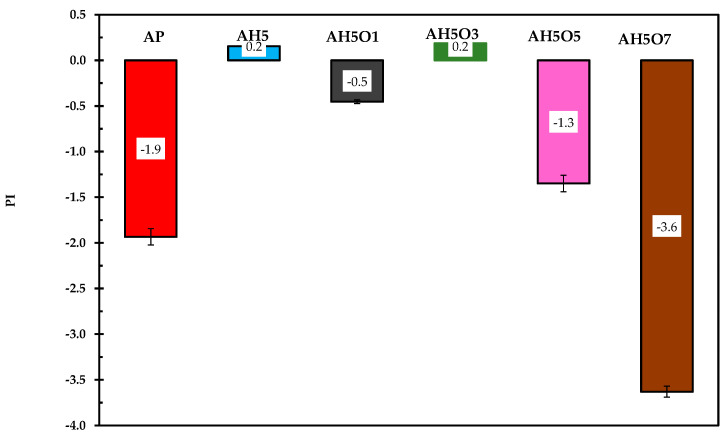
Penetration index indicating asphalt’s response to temperature variation.

The thermal susceptibility index (PI) represents the measure of asphalt’s response to temperature variations. Asphalt sensitivity was determined by PI using the penetration and softening point values. Asphalt is a temperature-sensitive material, showing diverse properties at different road service temperatures. According to Figure 4, the neat asphalt exhibited a PI value of −1.9, which shows that it is severely affected by temperature changes. After modification with 5 wt.% HDPE, the PI value increased, i.e., the HDPE/modified asphalt blend is less temperature susceptible.

The incorporation of soybean oil has a significant influence on the modified asphalt’s high-temperature performance. According to Lesueur [47], the PI value is a good indicator of the bitumen type, with PI > 2 being indicative of a gel bitumen, whereas PI < 0 is the value for a typical sol bitumen. Therefore, in the present study, the procedures used for asphalt modification did not change the internal structure of the binder. Adding to that, for paving purposes, PI values should range from −2 to +2. Thus, the PI values of the ternary blends of HDPE/soybean oil/asphalt are within the acceptable range for paving, except AH5O7.

### 3.4. Viscosity

**Figure 5:**
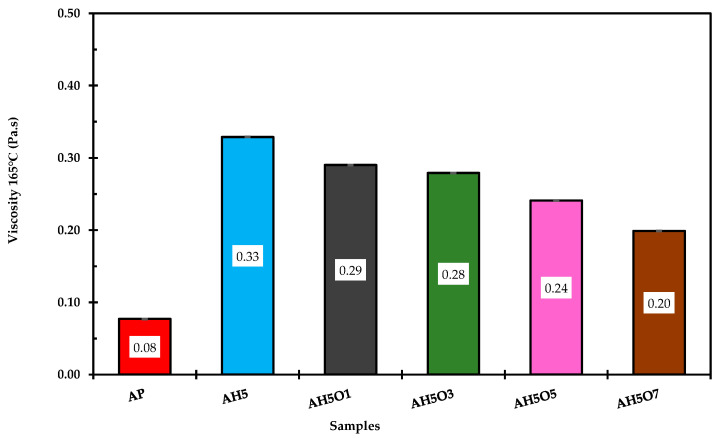
Viscosity test results of the asphalt binders at 165 °C.

Asphalt viscosity is a very important parameter for paving as it plays an important role in the asphalting process. Asphalt binders must remain sufficiently fluid or workable at high temperatures so that the energy required during the plant mixing, laydown, and compaction phases is minimized. The rotational viscometer measures the viscosity of the asphalt binder to evaluate its workability the during mixing and compaction processes. The effects of modifying asphalt binder with HDPE and soybean oil on the viscosity measurements at elevated temperatures using a rotational viscometer are shown in Figure 5.

These results show that the polymer-modified bitumen containing 5 wt.% of HDPE had a three-fold increase in its viscosity compared to the neat asphalt. At temperatures above 160 °C, HDPE is in a molten state, and it absorbs some oil and releases a low-molecular-weight fraction into the asphalt, which increases the viscosity of the modified asphalt. This result shows that HDPE may improve internal resistance of the base asphalt binder and prevent flow at high temperatures, consequently improving the resistance to rutting [48]. However, as stated by Joni et al. [12], although they exhibit better performance at high temperatures, the behavior against cracking of asphalt binders with high viscosity is weaker than that of low viscosity asphalt. Additionally, one of the most critical disadvantages of HDPE-modified asphalt mixtures is that the mixing/compaction stages should be carried out at very high temperatures of more than 40 °C higher than the mixing/compaction of asphalt mixtures with unmodified asphalt binder. On the other hand, the addition of soybean oil contributed to lowering the viscosity of asphalt binders, with a direct trend between the amount of soybean oil added and the viscosity of the corresponding asphalt binder. Accordingly, the addition of 1, 3, 5, and 7 wt.% reduced the viscosity at about 12, 15, 29, and 39% respectively, compared to the HDPE-modified asphalt binder.

Therefore, soybean oil improves modified asphalt workability due to lower viscosity, which in turn leads to lower mixing/compaction temperatures and increased resistance to cracking. According to Din and Mir [49], by decreasing mixing/compaction temperatures, less energy is required, less carbon dioxide and pollutants are released, and the aging of the binder is prevented by exposing the binder to less heat. Thus, the addition of soybean oil may facilitate the field compaction procedure of polymer-modified asphalt mixtures.

### 3.5. Ductility

**Figure 6:**
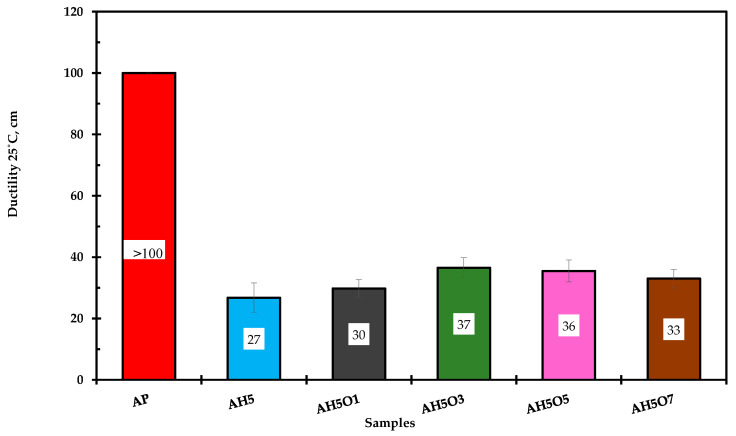
Ductility test results at 25 °C.

Ductility represents the extension or elongation ability of asphalt before fracture under tension. Asphalt pavement with high ductility has good durability. The ductility test was used to characterize the anti-cracking performance of asphalt. As can be seen in Figure 6, neat asphalt possesses an excellent extension property due to high ductility (not recorded since it was out of range of the ductility machine—100 cm).

When HDPE 5 wt.% was used as the modifier, the ductility of the modified asphalts showed an obvious decrease similar to the results obtained by Xiaoming and Eldouma [50]. The addition of soybean oil into the ternary composite binders induced a small ductility increase from 27 to 30–37 cm. These ductility values suggest that the modified binders became brittle and stiffer and may have better low-temperature performance, which might lead to moisture damage and low temperature cracking resistance compared with HDPE-modified asphalt. Similar results were reported by Rosyidi et al. and Wu et al. [51,52].

### 3.6. Effect of Thermal Oxidative Aging on Conventional Properties

**Figure 7:**
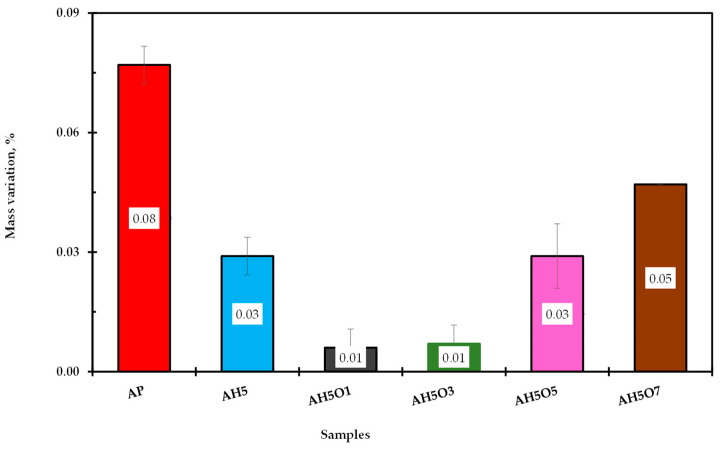
Mass variation due to aging process.

**Figure 8:**
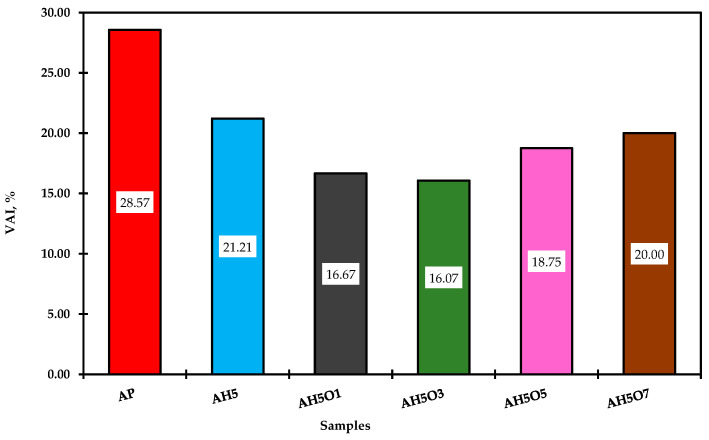
VAI results showing the antiaging propriety of the evaluated asphalt binders.

**Figure 9:**
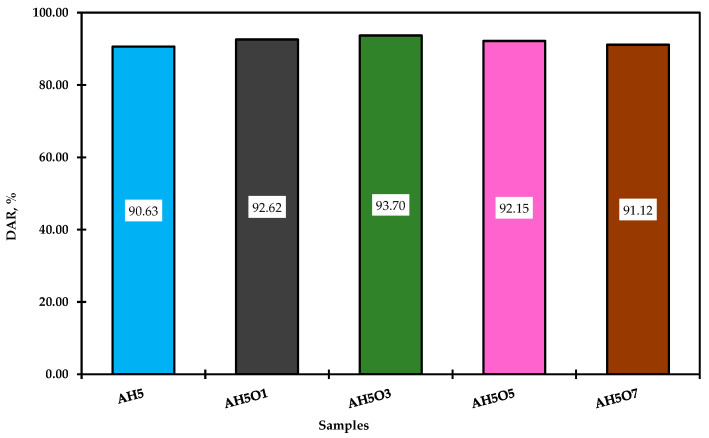
DAR results.

The asphalt samples were aged by RTFO, and the mass variation, the ductility at 25 °C, and the viscosity at 135 °C were all measured and compared with the properties of asphalt before aging. The mass variation, ductility aging ratio (DAR), and viscosity aging index (VAI) were computed and are presented in Figure 7, Figure 8 and Figure 9, respectively. It can be observed in Figure 7 that all samples gained mass after the aging process. According to Manoel [15], during this test two phenomena can occur: oxidation (mass gain) and volatilization (mass loss). Therefore, all studied binders showed some degree of oxidation.

The binder’s oxidation occurs first for the maltene fraction (aromatics), leading to formation of the asphaltene fraction; consequently, hardening and viscosity increase [53]. As reported by Tauste et al. [54], the oxidative aging is an irreversible diffusion-driven phenomenon controlled mainly by thermal reactions between atmospheric oxygen and asphalt components.

Nonetheless, these results show that the addition of 5 wt.% of HDPE results in reduction of the mass gain of neat asphalt by 63%. Further addition of 1 and 3 wt.% of soybean oil reduced the HDPE-modified asphalt mass gain by 67%. However, higher soybean oil content led to an increase in mass gain, showing induction to high oxidation when compared to binders with a lower content of soybean oil.

Although there was an increase in mass gain for binders with 5 and 7 wt.% of soybean oil, none of the modified binders showed high mass gain compared to the neat asphalt. Thus, simultaneous addition of HDPE and soybean oil up to 3 wt.% slowed down the aging of asphalt. The addition of soybean oil increased the ductility retention and decreased the viscosity aging index. As also observed by Zhang et al. [55], this higher ductility retention value and lower increment in viscosity (lower VAI) reflect less aging of the binders. Therefore, the addition of soybean oil up to 3 wt.% reduced the modified binder’s oxidation during short-term aging. As a result, these binders might suffer less when in contact with hot air or hot oxygen during the processing. For Tauste et al. [54], asphalt oxidation always yields a worse performance of asphalt mixture at low temperatures. Therefore, improving the aging properties of HDPE-modified asphalt by adding soybean oil leads to better performance at low temperatures and lower thermal cracking.

### 3.7. Thermogravimetric Analysis

**Figure 10:**
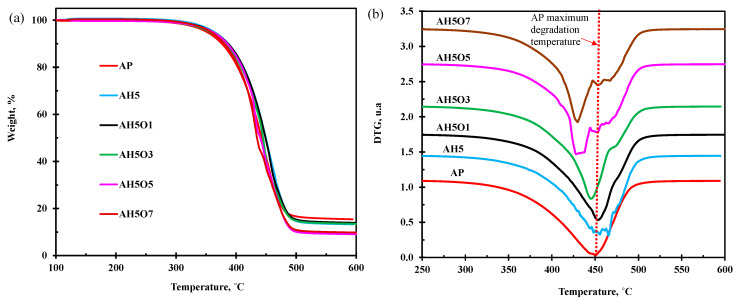
(**a**) TGA and (**b**) DTG curves for neat asphalt and samples of polymer- and oil-modified asphalt in N_2_.

In this study, the thermal stability of neat asphalt and the mixtures were studied by TGA in N_2,_ and the main characteristics of the TGA and DTG curves are presented in Table 3. Figure 10a illustrates the results of TGA of the samples of neat asphalt and asphalt modified with HDPE and vegetable oil. The data from the TGA curves reveals a similar behavior in terms of mass loss, and all exhibit a unique region of mass loss for virgin asphalt binder and HDPE-modified asphalt binder; however, DTG curves for the vegetable oil binder show that there are two partially overlapping reactions occurring in these binders. This finding agrees with results found by Ruiz [56].

Virgin asphalt has an initial degradation temperature of 304 °C and a residual mass of 15.42%. Considering that the tests were carried out in an inert atmosphere, the loss of mass is associated with evaporation or decomposition of the components [38]. The addition of the polymer raised the initial degradation temperature of the neat asphalt from 304 to 325 °C. The initial degradation temperature of the modified asphalt decreased as the concentration of vegetable oil increases. This behavior suggests a slight loss of the modified asphalt thermal stability due to the low thermal stability of the oil. The initial degradation varied with the amount of oil added and seemed to be directly proportional to the value of the thermal susceptibility index.

The addition of oil to modified asphalt shifts the maximum degradation temperature of the mixtures to lower values (Figure 10b).

HDPE-modified asphalt has higher molecular weight than vegetable soybean oil, and this results in decomposition of the polymer/asphalt-modified binder at higher temperatures than the asphalt/polymer/soybean oil composites [57,58]. The peak decomposition temperature decreased with increase in vegetable soybean oil due to low molecular weight and the presence of lightly weighted compounds and aromatic components in vegetable oil which make them less temperature resistant. On other hand, vegetable oil contains many oxygenated organic compounds, such as organic acids. When composites are heated up, the chemical composition of the vegetable oil changes toward thermodynamic equilibrium under special conditions, resulting in changes in the thermal stability. Although most carboxylic acids are relatively unstable, carbonation reactions occurred during thermal decomposition of binders, and decarboxylation formed the monoacid and CO_2_. The higher temperature and higher content of vegetable oil contributed to higher carbonation reactions and made the thermal resistance of triple composite performance decrease. However, modified binders do not become thermally degraded at actual storage and mixing temperatures (below 200 °C) or during the construction stage.

The results obtained by Grando [59] from the modification of asphalt with a high rubber content (22%) and different levels of used and contaminated lubricating oil indicate that the modification ended up being positive for the thermal stability of the samples since the maximum degradation temperatures were higher those of neat asphalt. However, from a practical point of view, it does not have a significant effect on thermal stability since the production and compaction temperatures of the asphalt mixture are lower than the approximate range of thermal decomposition (220 °C to 520 °C) of the binders. The high rubber content as well as the high thermal stability contributed to the increase in the maximum degradation temperature.

This is in agreement with results reported by Ruiz [56], where the authors attribute the first peak to the thermal decomposition of the main components of the bio-material, and the second one occurs due to the pyrolysis of the remaining chart residue.

## 4. Conclusions

This study evaluated the effect of two modifiers, HDPE and soybean oil, on asphalt’s physical, rheological, and thermal properties. The conclusions of the experimental work are summarized as follows:

- The addition of HDPE modifier to conventional asphalt improves the viscoelastic behavior of the asphalt and changes its rheological properties. The incorporation of 5 wt.% HDPE as a stand-alone additive reduces the penetration values and increases the softening point, the viscosity, and the processing temperature of the neat asphalt. On the other hand, this composition exhibited the least ductility, brittleness, and fracture before deforming much under a tensile load. The negative effect on the ductility values caused by the addition of HDPE can be mitigated with the addition of the vegetable oil.
- With the addition of vegetable oil, the penetration value of modified asphalt gradually increases and the viscosity decreases, which indicates that the oil makes the modified asphalt softer.
- The ternary blend with a combination of 5 wt.% HDPE and 3 wt.% soybean vegetable oil presented the best results, with low susceptibility to temperature variations, a penetration value close to the neat asphalt (penetration grade 50/70), as well as a low percentage of mass change (0.01%) when subjected to aging.
- Although the analysis conducted in this research provides a promising indication of the performance of HDPE- and vegetable-oil-modified asphalt, a more comprehensive evaluation is recommended with different tests, such as long-term performance, so as to evaluate the effect on storage, rutting, and cracking resistance under various traffic conditions. Further studies should also consider applying recycled HDPE and waste vegetable oil as an alternative recycling method for plastic and oil waste.

## Figures and Tables

**Table 1:** Neat asphalt properties.

Properties	Standard Test	Value	Specifications
Penetration (25 °C, 0.1 mm)	ASTM D5	54.0	50–70
Softening Point (°C, min)	ASTM D36	47.0	49–56
Ductility (25 °C, 5 cm/min)	ASTM D113	>100	min 100
Density (kg/m^3^)	D71 & D3289	990–1300	1010–1060
Flashpoint (°C)	ASTM D92	>230	min 230
Solubility (%, mass)	ASTM D2042	Soluble in most organic solvents	min 99.0

**Table 2:** Sample designation and composition.

Sample Designation	AP	AH5	AH5O1	AH5O3	AH5O5	AH5O7
HDPE, %	0	5	5	5	5	5
Soybean oil, %	0	0	1	3	5	7

AP—neat asphalt, AH—asphalt +HDPE and AHO—asphalt + HDPE + vegetable oil.

**Table 3:** TGA Results.

Samples		Temperature, °C		%Residue (25–60 °C)
	di	dmax	df	
AP	304	450	511	15.42
AH5	325	465	522	13.27
AH5O1	319	455	511	13.14
AH5O3	315	457	515	13.51
AH5O5	312	433	513	8.47
AH5O7	307	429	515	8.44

di—initial degradation temperature; dmax—maximum degradation temperature; df—final degradation temperature.

## Data Availability

The data presented in this study are available on requested from the corresponding author.
